# Strengthening intersectoral collaboration for adolescent sexual and reproductive health: a community-embedded intervention in Ebonyi state, Nigeria

**DOI:** 10.1186/s12978-025-01994-3

**Published:** 2025-05-31

**Authors:** Chinyere Ojiugo Mbachu, Irene Ifeyinwa Eze, Obinna Onwujekwe

**Affiliations:** 1 Health Policy Research Group, University of Nigeria Enugu Campus, Enugu, Nigeria; 2 Community Medicine Department, College of Medicine, Alex Ekwueme Federal University Ndufu-Alike Ikwo, Abakaliki, Nigeria

**Keywords:** Adolescent, Sexual and reproductive health, Intersectoral collaboration, Community-embedded intervention, Qualitative study

## Abstract

**Background:**

Adolescent sexual and reproductive health (ASRH) is a critical global health concern, demanding multifaceted approaches for effective intervention. This paper presents a qualitative exploration of an innovative community-embedded intervention aimed at enhancing intersectoral collaboration to improve ASRH outcomes.

**Methods:**

A qualitative study design was employed to explore the types, processes, and factors that enabled or constrained intersectoral collaborations in implementing a community-embedded intervention for ASRH in Ebonyi State, Nigeria. Thirty in-depth interviews and 18 focus group discussions were conducted with policymakers, health service providers, teachers, community gatekeepers, parents, and adolescents. The interview transcripts were coded in NVivo 12. The outputs of the coded transcripts were analyzed using thematic analysis and presented as narratives.

**Results:**

Key findings highlight the emergence of three predominant collaboration models: horizontal integration between healthcare and education sectors, horizontal integration across various governmental agencies (formal providers) and non-governmental organizations (informal providers), and diagonal integration bridging gaps between community stakeholders and service providers. The processes underpinning these collaborations emphasize the significance of trust-building, shared goals, and clear communication. The study identifies the pivotal role of champions and intermediaries in facilitating collaboration, along with the necessity for a supportive institutional framework. The enabling factors include robust leadership commitment, dedicated funding mechanisms, and a favorable policy environment. Conversely, challenges stemming from resource constraints, conflicting interests, and organizational silos hindered collaboration efforts.

**Conclusion:**

This research underscores the potential of community-embedded interventions to strengthen intersectoral collaboration for the acceptability and adoption of ASRH strategies. It offers a valuable resource for policymakers, healthcare professionals, and stakeholders seeking to enhance ASRH outcomes through collaborative efforts.

## Plain Language Summary

This research focuses on improving adolescent sexual and reproductive health (ASRH) in Ebonyi State, Nigeria, through a community-embedded intervention. The study explores how different groups like policymakers, healthcare providers, teachers, community leaders, parents, and adolescents work together to improve ASRH.

The study found three main ways these groups collaborate:

1. Horizontal collaboration between healthcare and education sectors.
2. Horizontal collaboration between government agencies and non-governmental organizations
3. Diagonal collaboration bridging gaps between community members and service providers.

Successful collaboration relies on building trust, having shared goals, and clear communication. The study highlights the importance of champions and intermediaries who help facilitate collaboration. It also emphasizes the need for strong leadership, dedicated funding, and supportive policies to make these collaborations work.

However, challenges like limited resources, conflicting interests, and organizational barriers can make collaboration difficult. Overall, the research shows that community-embedded interventions can improve ASRH by bringing different groups together to work toward a common goal.

## Résumé

**Contexte:**

La santé sexuelle et reproductive des adolescent(e)s et jeunes (SSRAJ) est un problème de santé mondial crucial, exigeant des approches multidimensionnelles pour une intervention efficace. Cet article présente une exploration qualitative d’une intervention innovante communautaire, visant à renforcer la collaboration intersectorielle pour améliorer les résultats de la SSRAJ.

**Méthodes:**

Une étude qualitative a été réalisée pour étudier les types, les processus et les facteurs qui ont permis ou limité les collaborations intersectorielles dans la mise en œuvre d’une intervention communautaire pour la SSRAJ dans l’État d’Ebonyi, au Nigéria. Trente entretiens approfondis et 18 discussions de groupe ont été menés avec des décideurs politiques, des prestataires de services de santé, des enseignants, des responsables communautaires, des parents et des adolescents. Les transcriptions des entretiens ont été codées dans NVivo 12. Les résultats des transcriptions codées ont été analysés à l’aide d’une analyse thématique et présentés sous forme de récits.

**Résultats:**

Les principales constatations mettent en évidence l’émergence de trois modèles de collaboration prédominants, à savoir l’intégration horizontale entre les secteurs des soins de santé et de l’éducation, l’intégration horizontale entre divers organismes gouvernementaux (prestataires officiels) et organismes non gouvernementaux (prestataires informels) et l’intégration diagonale comblant les écarts entre les parties prenantes de la communauté et les prestataires de services. Les processus qui sous-tendent ces collaborations soulignent l’importance de l’instauration de la confiance, d’objectifs communs et d’une communication claire. L’étude identifie le rôle central des champions et des intermédiaires dans la facilitation de la collaboration, ainsi que la nécessité d’un cadre institutionnel de soutien. Les facteurs habilitants comprennent un engagement solide des dirigeants, des mécanismes de financement dédiés et un environnement politique favorable. À l’inverse, les défis découlant des contraintes de ressources, des intérêts conflictuels et des cloisonnements organisationnels ont entravé les efforts de collaboration.

**Conclusion:**

Cette recherche souligne le potentiel des interventions communautaires de renforcer la collaboration intersectorielle pour l’acceptabilité et l’adoption des stratégies en matière de SSRAJ. Elle offre une ressource précieuse aux décideurs politiques, aux professionnels de la santé et aux parties prenantes qui cherchent à améliorer les résultats de la SSRAJ par le biais d’efforts de collaboration.

## Background

Adolescent sexual and reproductive health (ASRH) remains a critical global public health concern, particularly in regions like sub-Saharan Africa, where myriad socio-cultural, economic, and healthcare system challenges can impede comprehensive and effective interventions [1–3]. Community norms [2] and economic factors, such as poverty and lack of financial resources, can hinder adolescents' access to reproductive health services such as contraceptives, antenatal care, and safe abortion services where they are legal [1–3]. Weaknesses in healthcare systems, including inadequate infrastructure, healthcare workforce shortages, and limited availability of youth-friendly services, can impact the delivery of sexual and reproductive health (SRH) services to adolescents [4–6].

Empirical evidence indicates that sub-Saharan Africa has some of the highest rates of adolescent pregnancies and HIV/AIDS among young people, globally. The World Health Organization (WHO) reported in 2018 that the region had the second-highest adolescent birth rate, with approximately 101 births per 1000 adolescent girls [7, 8]. A significant proportion of new HIV infections occur among young people (15–24 years) in sub-Saharan Africa [9]. Many countries in the sub-region face challenges in providing comprehensive sexuality education to adolescents [10, 11]. The UNESCO International Technical Guidance on Sexuality Education emphasizes the importance of age-appropriate, culturally relevant, and scientifically accurate sexuality education [12, 13]. However, implementation varies, and there are often gaps in accessibility and quality [14, 15].

Furthermore, West Africa has enormous work to do to ensure equitable access to adolescent health services across the region. Despite improvements in recent years, West Africa has the most challenging health indicators globally, particularly regarding sexual and reproductive health [16]. The region has the highest adolescent fertility rate globally—128 per 1000 women, and adolescents make a significant contribution (13 percent) to maternal mortality and disabilities (obstetric fistula). While the HIV prevalence rates may seem low (two percent) the absolute numbers are high—almost one-third of the 25 million people in sub-Saharan Africa living with HIV are in this region, with women being disproportionately more affected compared to men. The contraceptive rate in most of the countries is under 15 percent, while unmet needs are high. Only 48 percent of pregnant women are assisted by skilled personnel at birth [17]. In addition, the region faces a major challenge with insufficient skilled health personnel to meet the demand for health services. Ensuring sufficient skilled human resources with the necessary skills mix to address current and emerging health issues in an enabling environment is critical for building the resilience of health systems.

Nigeria, as the most populous country in Africa, grapples with the complexities of addressing the unique health needs of its adolescent population. The country’s demographic landscape is characterized by a significant youth bulge, with adolescents comprising a substantial proportion of the population—about 70% of the population under 30 and 42% under the age of 15 [18]. Despite this demographic reality, ASRH faces multifaceted challenges rooted in cultural norms, limited access to comprehensive sexuality education, and barriers to reproductive health services [1–3]. Furthermore, persistent gender inequalities exacerbate vulnerabilities among adolescents, hindering their ability to make informed decisions about their sexual and reproductive health [19–21]. Compounding these challenges, traditional healthcare structures often operate in silos, lacking coordination and collaboration across sectors [22]. This fragmentation has impeded the effectiveness of previous interventions, necessitating a paradigm shift towards a more integrated and community-embedded approach that will promote strategic alliances that transcend traditional health boundaries [23].

Intersectoral collaboration involves engaging diverse stakeholders, including healthcare professionals, educators, community leaders, and policymakers, to collectively address the multifaceted determinants of health [23]. Intersectoral collaboration is recognized as a strategy for addressing the social determinants of health. The WHO Commission on Social Determinants of Health highlighted the importance of collaboration between health and other sectors to tackle the root causes of health inequities. This approach involves actions beyond the health sector, including education, housing, and employment, to improve overall well-being [23]. Intersectoral collaboration for health has been implemented in various contexts, and several studies highlight the potential benefits of improving health outcomes [24–26]. This includes better access to, and utilization of health services among vulnerable populations, and a reduction in health inequalities [24, 27].

Community-embedded interventions, which often involve intersectoral collaboration, have shown promise in improving health outcomes [28–30]. This is particularly necessary for ASRH because access to and utilization of SRH services by adolescents are influenced by social norms around premarital sex and gendered sexual norms [31]. Community-embeddedness, which is a central tenet of norm change, emphasizes the active involvement and empowerment of local communities. It acknowledges the diversity of communities by tailoring interventions to the specific needs, beliefs, and practices of each locale. By engaging community members as partners rather than passive recipients of healthcare initiatives, existing social structures, cultural practices, and community leaders can be leveraged to amplify the impact of ASRH interventions. Community members become active agents of change, fostering a sense of ownership and sustainability of health interventions.

Ebonyi State, situated in the southeastern part of Nigeria, reflects the broader challenges facing the nation in addressing ASRH. In this context, a community-embedded intervention was implemented, leveraging intersectoral collaboration to foster an ecosystem that integrates health, education, and community engagement [21]. The overarching goal of our intervention was to improve ASRH outcomes among adolescents in Ebonyi State by strengthening intersectoral collaboration in providing SRH information and services to adolescents. The anticipated implementation outcomes included the acceptability and adoption (by all stakeholders) of an intersectoral approach to delivering SRH information and services to adolescents. Therefore, the intervention aimed to create a sustainable framework for continued intersectoral collaboration that would contribute to better access to SRH information and services for adolescents.

This research offers a nuanced understanding of intersectoral collaboration dynamics in the context of ASRH. This paper sheds light on the various types of collaborations formed, the intricate processes involved in creating and/or revitalizing these collaborations, and the influential factors that either enabled or constrained these partnerships.

## Methods

### Study design and setting

This is a qualitative study conducted through in-depth interviews (IDI) and Focus Group discussions (FGDs) in Ebonyi State, southeast Nigeria. The study aimed to shed light on the various types of collaborations formed for ASRH, the intricate processes involved in creating and/or revitalizing these collaborations, and the influential factors that either enabled or constrained these partnerships. The State was selected because of the poor ASRH indices -teenage pregnancy [32]. Ebonyi State has an estimated population of 2.9 million as of 2016, a fertility rate of 5.4, and an adolescent birth rate of 107/1000 [32]. The State has 13 local government areas (LGAs) stratified into three senatorial zones. Six LGAs (two per senatorial zone) were purposively selected for this study; the state government has prioritized these LGAs for SRH interventions due to the high teenage pregnancy rate [33]. From the selected LGAs, six communities (one per LGA) that had received a package of community, PHC, and school-based SRH interventions to improve access to SRH information and health services for adolescents served as the study sites. Thus, a total of six communities, six primary health centres (PHC), and six secondary schools participated in the study.

Health service delivery in the State is structured into a three-tier system: the primary level of care, the secondary, and the tertiary levels, which render sexual and reproductive health services. The State is mostly rural and is inhabited predominantly by Christians.

### The SRH intervention

The community-embedded intervention involved multisectoral collaboration of numerous stakeholders to design and implement a multi-faceted intervention that was simultaneously implemented at several sites—health facilities, schools, and communities. The stakeholders were policymakers, health facility managers and service providers, community health workers (CHWs), patent and proprietary medicine vendors (PPMVs), school principals and teachers/guidance counsellors, peer educators and community leaders, parents, and adolescents. The components and objectives of the intervention were: (i) stakeholder engagement to gain support and buy-in of the intervention through targeted advocacy visits to policymakers and community leaders/citizen consultations; public panel discussion; policy dialogues and distribution of policy briefs and facts sheet (ii) capacity building of the state trainers, health facility managers and service providers, CHWs and PPMVs through three-days step-wise training to build/enhance their capacity on the provision of adolescent-friendly SRH services, and supportive supervision (iii) training of school teachers/guidance counsellors and peer mentors via three-day workshops and establishment of school-based youth clubs to enhance their capacity for the provision of comprehensive sexuality education, (and reinforced with the distribution of ASRH customized sensitization items—notepads, fliers shirts, caps, wrist bands, pens), (iv) sensitization and awareness creation of community gatekeepers, parents, and adolescents on SRH and rights using the above tools. Further details of the intervention can be found in an earlier published manuscript [21].

### Study population and sampling

The study population included stakeholders from the health sector (Ministry of Health and State Primary Health Care Development Agency -SPHCDA), boundary partners, and community members. The boundary partners here refer to stakeholders working in government sectors relevant and/or involved in adolescent SRH such as Ministries of Education, Information, Youth Development, and Women Affairs. Thus the study population comprised appointed and career policymakers from Ministries of health, education, information, youth development, and women affairs, health service providers at the primary health centres (PHCs), including adolescent focal persons, officers in charge (OIC) of PHCs, CHWs, and PPMVs; school officials—principals, teachers, guidance counsellors; and community members including traditional leaders, community heads, parents; and adolescents—both in and out of school aged 13–19 and youth advocates across the six intervention clusters. The qualified participants who provided written informed consent were purposively selected for inclusion in the study. Participants who were indisposed to communicate due to severe or debilitating medical conditions by the scheduled date for data collection were excluded from participating.

The participants for the IDIs comprised 16 career and appointed policymakers, eight adolescent focal persons and health administrators, and six school administrators (principals, teachers, and guidance counsellors). The participants for the FGDs comprised healthcare providers (PHCs OICs, CHWs, and PPMVs) and community members (community leaders, parents, and adolescents disaggregated by designation and sex. Details of participant distribution are presented in Table 1 below.

**Table 1:** Distribution of study participants

Description of participants	Type of interview	
	No. of IDI	No. of FGD
Policymakers and boundary partners		
State Ministry of Health	3	–
State Ministry of Education	1	–
State Ministry of Information	1	–
Legislator	1	–
State Ministry of youth and sports development	1	–
Primary Health Care Development Agency	1	–
Media (Ebonyi State Broadcasting Corporation)	2	–
SDGs	1	
Traditional rulers	1	–
Religious leader	1	–
NGO	1	–
CSO	I	–
Health and school service providers		
LGA ASRH focal officers	6	–
LGA admin secs	2	–
Principals/GC teachers	6	–
PHC workers OICs	–	2
PMVs	–	2
CHWs	–	2
Parents, community leaders and adolescents		
Male parents	–	1
Female parents	–	1
Male community leaders	–	1
Female community leaders	–	1
Male in-school adolescents	–	2
Female in-school adolescents	–	2
Male out of school adolescents	–	2
Female out of school adolescent		2
Total	30	18

### Data collection methods

The study was conducted from April 2021 to August 2022 through 30 in-depth interviews (IDI) and 18 Focus Group discussions (FGDs) with stakeholders involved in ASRH. Participants for the IDIs included all the stakeholders who were involved in delivering the interventions in the six communities and schools. For the FGDs, participants were randomly recruited from the various groups that participated/benefitted from the interventions.

Semi-structured interview/topic guide was developed by the researchers to examine three key constructs (i) types of intersectoral collaboration, (ii) processes of intersectoral collaboration comprising stakeholder engagement through advocacy, public panel discussion, policy dialogues, distribution of policy briefs, facts sheet, and educational materials, capacity building and supportive supervision of the stakeholders, and (iii) barriers and enablers of intersectoral collaboration. The topic guides explored respondents’ perspectives and experiences with the intervention in subject areas. Prompts were included to ensure that all the relevant aspects of the research questions were exhausted.

The interviews/discussions were conducted face-to-face from September 2021 to December 2021 by six social scientists (male and female) who were identified as having experience in conducting qualitative interviews and managing the data and independent from the implementation team were recruited and trained. The training was to acquaint them with the research project and ensure a uniform understanding of the topics and questions in the interview guides and the ethical principles in research (including researching minors). The participants were approached through invitation letters and scheduled appointments, visits, telephone calls, and text messages. Written informed consent for participation and digital recording of the interviews/discussions was sought from interviewees before the interviews were initiated. Personal identifiers were redefined to protect the participants' identities, maintain anonymity, and facilitate confidentiality. The IDIs were conducted individually for the selected stakeholders at their respective offices. The FGD, which comprised 6–8 persons in each group, was conducted in quiet and convenient locations chosen by respondents. The interviews were either conducted in English language or the local language according to the preference of the participants. Observation and notes taken during the interview and at the SRH intervention meetings were used to gain a deeper understanding of operational issues and the context in which the intervention was conducted. The FGD lasted 60–90 min, and the IDI 45–60 min.

### Data management and analysis

The audio recordings of the interviews were transcribed verbatim, translated into English, and reviewed with the notes taken during the interview. Each transcript was read by the researcher who facilitated/moderated the interview/discussion to ensure that the data was completely and accurately captured before subjecting the transcript to analysis.

**Fig. 1:**
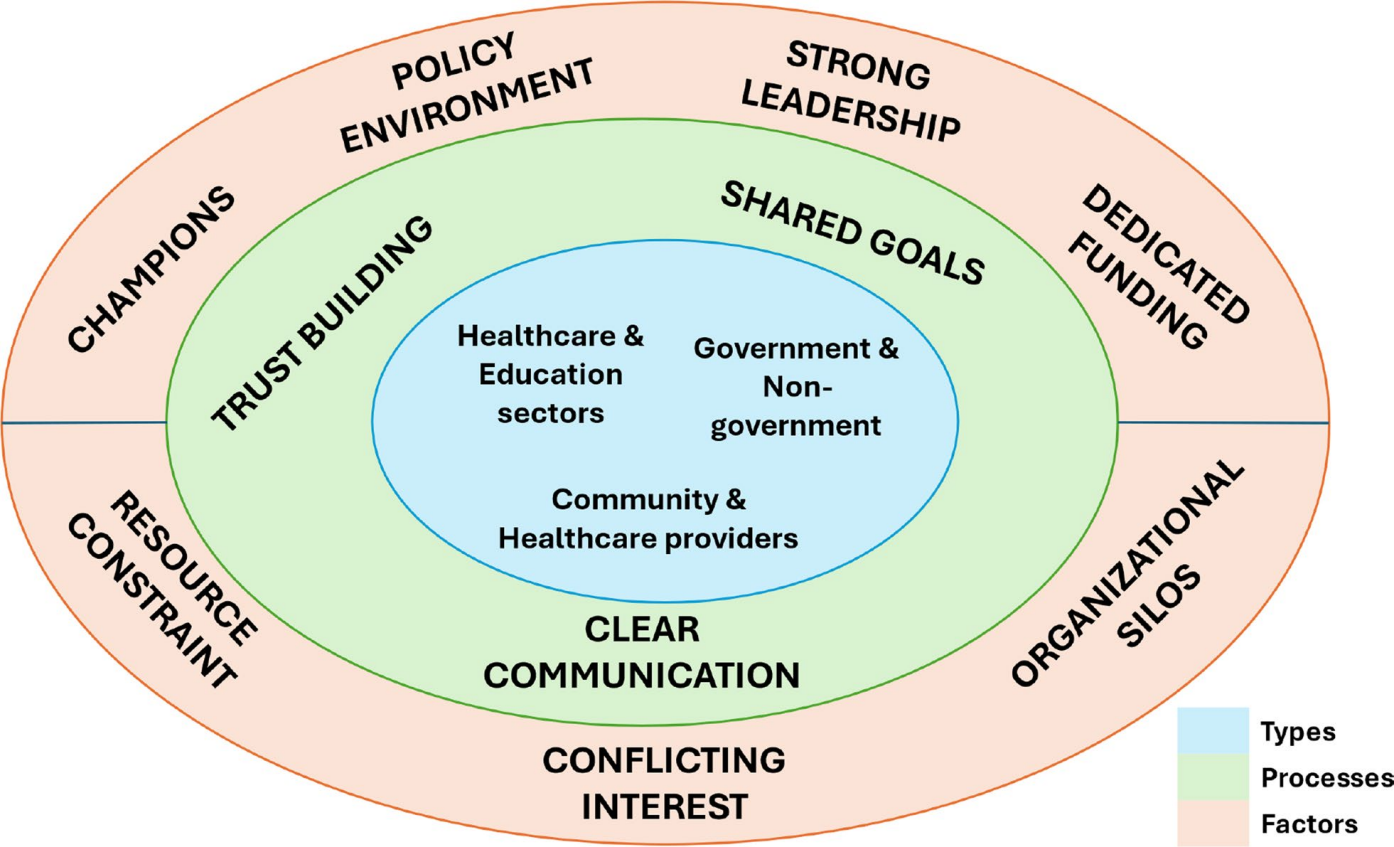
Framework of types, processes, and factors influencing intersectoral collaboration

Inductive thematic analysis was conducted. Inductive coding was used to explore themes as they emerged [34, 35]. Three transcripts were read through for familiarization and coded manually by three researchers. A senior social scientist also coded some of the initial transcripts and compared notes with the researchers to ensure coding consistency and comparability and to facilitate collaborative thematic analyses throughout. The transcripts were then imported into NVivo 12 software, Texas, USA, for analysis. The generated themes and sub-themes were continually reviewed and refined to capture emerging codes. The emerging themes for strengthening intersectoral collaboration for adolescent sexual and reproductive health using community-embedded intervention revealed interactions between healthcare and education sectors, government and non-government, and between community and healthcare providers driven by trust building, clear communication, shared goals as well as leadership and governance, as shown in Fig. 1. Quotes were captured to highlight the thematic areas and increase our understanding of the context.

## Results

A total of 143 persons participated in the study, comprising policymakers 16 (11.4%), health services providers 47 (33.3%), teachers 6 (4.3%), community leaders and parents 24 (17%), and adolescents 48 (34.0%). Majority of the participants, 80 (56.7%), were females, and 61 (42.3%) were males.

Details of the results presented under themes and sub-themes are shown in Table 2.

**Table 2:** Stakeholders’ perspective of types, processes, and factors influencing intersectoral collaboration

S/No	Theme	Sub-theme
1	Types of collaboration	i. Horizontal integration across government agencies
		ii. Horizontal integration between formal providers (government agencies) and informal providers (non-government agencies)
		iii. Diagonal integration between community stakeholders and service providers
2	Processes of collaboration	i. Trust-building ii. Shared goals iii. Clear communication
3	Factors that enable or constrain collaboration	i. Facilitators
		(a) Availability of champions and intermediaries
		(b) Favorable policy environment
		(c) Leadership and commitment
		(d) Dedicated funding mechanisms
		ii. Barriers
		(a) Resource constraints
		(b) Conflicting interests
		(c) Organizational silos

### Types of collaboration

Respondents reported multi-sectoral collaboration with multiple stakeholders including (i) horizontal integration across government agencies, (ii) horizontal integration between formal providers and informal health providers, and (iii) diagonal integration between community stakeholders and health service providers. The horizontal integration highlighted the collaboration of stakeholders across government agencies, notably health and education sectors—both secondary and primary education boards, as well as other sectors like Ministries of Information, Youths’ development, and Women affairs. Furthermore, there was horizontal integration between formal (government) health providers—(policymakers from the Ministry of Health and state primary health care development agency, health facility managers, and service providers) and informal health providers—community health workers, patent and proprietary medicine vendors. The diagonal integration entailed collaboration between the health service providers and community stakeholders, including traditional leaders, community/village heads, parents, and adolescents, to bridge gaps.

#### Horizontal integration across government agencies

Respondents reported collaboration between healthcare and various government agencies like education, youths’ development, and women affairs. Notable in the collaboration, is between the health and education sectors in implementing a variety of activities, including training of teachers, guidance counsellors, and peer educators/mentors, instituting a school health club, supportive supervision, and sensitization on SRH matters. “*The collaboration between the education and health sector is robust. They include establishing and inauguration of school health clubs, training of teachers and peer educators; and the peer learning is worthwhile”* (IDI Boundary Partner-2, Female).

Respondents also acknowledged that the horizontal integration, which utilized an intervention co-creation strategy, provided an opportunity for stakeholders to brainstorm, share knowledge, and develop relevant and context-specific interventions to address adolescents’ sexual health needs and problems. “…*the approach brought us together to work as a team, sharing knowledge and learning from each other”.* (IDI, career policy maker-2, Female).

#### Horizontal integration between formal providers (government agencies) and informal providers (non-government agencies)

It was also noted by respondents that there were collaborations between formal providers in government primary health centres and the informal providers (specifically PPMVs and CHWs) in delivering SRH services to adolescents in the communities and knowledge sharing. The health providers described the training of the community health volunteer workers and patent medicine vendors, who are the first point of call for care, especially in the rural communities, as worthwhile as it enhanced their capacity to provide appropriate information and services to adolescents. While expressing the gains of the integration, a respondent stated *“The training helped me much. I have been using it for my customers, and it is working.”* (FGD, PPMV-1, Male).

#### Diagonal integration between community stakeholders and service providers

The respondents expressed that the diagonal integration helped bridge the gaps between community stakeholders and service providers and address social norms that constrain access to SRH services and the provision of SRH services. They viewed the sensitization of the community leaders/members, including parents and young persons, as a welcome development that enabled a better understanding of issues concerning adolescent health. “*I have been using the information I got from the training to educate my younger ones not to continue bad lifestyle* (risky sexual behaviour)” (FGD, Adolescent-4, Female).

It is noteworthy that the stakeholders viewed the community-embedded intervention strategies to strengthen intersectoral collaboration as innovative, describing the intervention in relation to the source, effectiveness, relative advantage, adaptability, and design. Related to the source, they expressed that the group that facilitated the intervention was from a university/research institution that is reputable and credible, as well as trustworthy policymakers from the state ministries.*“…be assured that the intervention is of quality. You know, the manuals were developed by university academicians and renowned policymakers”*. (IDI, career policy maker-1, Female)*.* They added that the collaboration strategy had robust results supporting its effectiveness, such as creating numerous avenues to reach many adolescents “…*different types of adolescents can now be reached for their sexual health needs at different places.”* (IDI, Religious leader-1, Male). In referring to their professional responsibility, stakeholders expressed that the intersectoral collaboration had a relative advantage compared to their previous practice of working in silos as it promoted mutual relations to build trust. A community influencer stated, “…*most parents lacked the skill to discuss sexual matters with young persons. The coming together of experts, community people, and young people created a bond of trust among the stakeholders.”* (IDI, Community Influencer-2, Male). In addition, respondents pointed out that the intersectoral collaborations enabled the intervention to be well-designed and packaged in a manner that can be easily modified to fit local contexts or needs. To buttress their view, they highlighted the positive effect of the stepwise training model whereby the tools were adapted to the different levels of stakeholders using multiple but simple and suitable training modes and formats to include enhancing sustainability. “*The training manual is friendly as it is simple and handy, unlike the national training manual.” (*IDI, career policy maker-2, Female).

Respondents expressed that the intersectoral collaboration revealed multiple levels of setting involvement: system, state, and community, including the local attitude through the involvement of stakeholders in government establishments, non-governmental organizations, and communities, including traditional and religious leaders. It allowed looking into sociocultural values (e.g., shared responsibility in helping recipients of SRH services) and beliefs (e.g., convictions about the worthiness of recipients) and encouraged the community to support the implementation and delivery of the intervention. A respondent stated, “…*it has affected our way of life and the norms. Before, they believed that area* (SRH) *is a go area, and we should be ‘silent (*not have an open discussion*), but that feeling has changed” (*IDI, community influencer -2, Male)*.* They added that it gave route to the use of societal pressure like mass media campaigns, advocacy groups like civil society organizations, and social groups (e.g., community and church organizations), which helped drive the implementation and delivery of the intervention “*With the support from different stakeholders, I now see that most of the girl children now voluntarily go for counselling in some of our health facilities”* (IDI, CSO-1, Male).

### Processes of collaboration

Findings showed that successful collaborations relied on three processes – trust-building, shared goals, and clear communication.

#### Trust building

The stakeholders reported that they joined together, intentionally coordinating and collaborating on interdependent tasks, to implement the intervention because they believed, valued, and trusted each other. A respondent stated, “…*meeting and interacting with varied experts and specialists to improve adolescents’ health made me feel valued and motivated to participate.” (*IDI, Boundary partner-4, Female).

#### Shared goals

They pointed out the pivotal role of planning in the process, where roles and responsibilities were identified, outlining specific steps and milestones, defining goals and measures for implementation success in advance, and going further to tailoring the strategies by choosing and operationalizing implementation strategies to address barriers, leverage facilitators, and fit context. A respondent summarized the process in this way. The respondents expressed that the intersectoral collaboration enabled stakeholders with unique roles to come together and share their knowledge and skill; thus, synergy in addressing adolescent SRH matters, adding that the collaboration helped them to interact and agree on common achievable goals “…*you cannot handle adolescent sexual issues alone as an individual or a sector, you need multiple stakeholders to be involved to agree on the best line of action.”* (IDI, career policy maker-2, Female).

#### Clear communication

The processes underpinning these collaborations emphasize the significance of need and context assessment, which enabled a good understanding of the situation. They highlighted the importance of the need and context assessment employed in the process of implementation, which entailed collecting information about priorities, preferences, and needs of the deliverers and recipients as well as identification and appraisals of barriers and facilitators to implementation and delivery to guide the formulation of policies and practice. They added that the production and use of policy briefs and fact sheets, and reflection and evaluation processes, which involve collecting and discussing quantitative and qualitative information about the success of implementation and/or the intervention, were noteworthy. “…*from the initial evaluations that were shared, we know and understood our problems and so, we can plan well towards solving them.*”* (*IDI, career policymaker-2, Female).

Approaches that were noted to help revive dormant collaborations include stakeholder engagement, capacity-building, and policy alignment.

Stakeholder engagement: The processes of creating and reviving dormant collaborations were found to require proactive efforts, often involving wide stakeholder engagement, one-on-one advocacy, citizen consultations, and stakeholder dialogues which were engaging, as deliverers and recipients were attracted through well-focused and directed advocacy and encouraged to serve on the implementation team and/or participate in the intervention. A respondent stated, “…*different specialists came together and brought varied ideas on how to improve adolescents’ health. We had robust discussions and learned from each other, which made me feel valued and motivated to participate.” (*IDI, CSO-1, Male).

Capacity building: Respondents expressed that the intersectoral collaboration enabled individuals with high levels of competence, knowledge, skills, and subject matter expertise willing to assist, coach, or support implementation to come together and facilitate the process, adding that the stakeholders knew what to do and whom to call when the need arose. An appointed policymaker stated, *“I am happy that there are many hands to learn from and work with on adolescent health issues.”* (IDI, appointed policymaker-1, Female). A health provider added “*The training has helped me much to improve in my work.”* (FGD, Service provide-3, Female). Furthermore, there was the expression of pleasure towards the gains of the supportive supervision that was provided which enabled coaching to support service provision. A respondent asserted this fact by saying *“…the health workers at the PHC are willing to listen to us and provide guidance. They even come for supportive supervision.”* (FGD, PPMV-1, Male)*.* Another confirmed the assertion when she stated “…*it is part of our work, to train and provide supportive supervision for the service providers at the PHC. With the training, we improved in our work” (*IDI, career policymaker-1, Female).

Policy alignment: Respondents pointed out that the implementation process was facilitated by favourable policy alignment in terms availability of policies and laws, legislation, and regulations that support implementation and/or delivery of the intervention both in the formal and informal sectors, as expressed by a carrier policy maker “…*the training manual was adapted from the national training manual.” (*IDI, career policymaker-2, Female).

### Factors that enable or constrain partnerships.

#### Facilitators

The enabling factors stated by the respondents include the availability of champions and intermediaries, robust leadership commitment, dedicated funding mechanisms, and a favourable policy environment.

Availability of champions and intermediaries: The study identified the pivotal role of champions and intermediaries in facilitating collaboration, along with the necessity for a supportive institutional framework. Noteworthy is the positive effect of engagement of varied stakeholders, including the traditional institutions, and the use of champions as deliverers and recipients of the intervention. The benefits of utilizing institutional frameworks such as institutionalizing sexuality education through its incorporation into the school curriculum and the expression of the corporate responsibilities of media stakeholders by using the mass media in awareness creation were pointed out. “*This gain was expressed when she stated “We now understand issues concerning adolescents and especially their sexual matters better. Now, our GM is championing adolescent issues, our media outlet has granted slots to discuss adolescent health*. “ (IDI, Boundary partner-5, Female).

Leadership and commitment: The respondents expressed that the intervention benefited from robust leadership commitment evidenced by the presence of individuals with a high level of authority from University, State, and local government, including key decision-makers, executive leaders, directors, and community influencers with both formal and informal influence on the attitudes and behaviours of others. According to them, most of the implementers were individuals with subject matter expertise who were willing and eager to assist, coach, or support the implementation and had interpersonal competence, knowledge, and skills to tackle tasks. In addition, the individuals have the availability, power, commitment, and motivation to fulfil the role. “*You can see the calibre of people involved in the project—*career *and appointed policymakers, the leadership of the religious and traditional institutions, and the rest, it shows the high level of interest to succeed*.* (*IDI, career policymaker-1, Female).

Dedicated funding mechanisms: Respondents expressed that they leveraged the vast number of implementing partners, attracted by the supportive environment in the state, to fund SRH services both for the health and non-health sectors. The networking, partnership, and connection with external entities, including referral networks, academic affiliations, and professional organization networks, facilitated the processes. A respondent stated “*We are just blessed in Ebonyi state; many implementing partners are supporting the project with funding. (*IDI, career policymaker-2, Female).

Favourable policy environment: Worthy of note is the expression of a favourable policy environment. It was further noted that the policies and guidelines in place aligned well and fit with their roles and responsibilities, thus making the implementation of the intervention less burdensome. A health worker stated, “*It’s our work schedule, they have just helped us to improve and make it easier.” (*FGD service provider-2, Female).

#### Barriers

Challenges that stemmed from resource constraints, conflicting interests, and organizational silos were enumerated as hindering collaboration efforts.

Resource constraints: Respondents lamented the insufficient human resources to implement the intervention despite the collaboration with some groups, highlighting the need to identify and collaborate with more stakeholders to enable adequate attention to be given to adolescent sexual and reproductive health needs. A community influencer stated, “*…more hands are needed. We cannot underestimate the sexual and reproductive health needs and negative health outcomes of these young persons.”* (IDI, Community Inluencer-1, Male).

Organizational silos: Respondents pointed out that operating in organizational silos is one of the major barriers to penetrating adolescents with the need for SRH services, as different sectors and stakeholders have different levels of awareness, capacity, and biases toward addressing adolescents' sexual health needs. They emphasized that incorporating sectors should not be limited, as enlarging the scope to include many relevant government ministries and agencies, mass media, community settings, voluntary organizations like Girls Guides and Boys Scouts, etc., will be tantamount to better results. “*This program has not spread enough. Am suggesting that this program be taken to different churches, where many people will be around and listen to it. Although many people have benefited from it, more awareness needs to be created in different churches and remote villages, and if possible, involve the government people so that they will not be afraid.” (*FGD, Service provide-3, Female). Another respondent buttressed the need not to work in silos by expressing, “*You see, the tree cannot make a forest. This thing should be a teamwork. Everybody who matters to be on this project for us to achieve our aim should be carried along, both from the Ministry of Information, Health, and Women Affairs. They should be there, so it is important to collaborate with all of them. It should be a collective responsibility.” (*IDI, career policymaker-2, Female).

Conflicting interests: This was expressed by stakeholders in terms of different areas of focus and shared responsibilities as part of the challenges affecting SRH service provision and delivery. They, however, expressed the need for a good relationship and defined roles and responsibilities to avert conflicting interests. “*We need good collaboration between groups to be able to have a steady supply of commodities. Otherwise, when these adolescents in the community want to collect condoms, they will be told not to use them.” (*IDI, Boundary partner-1, Female).

## Discussion

This research highlights three main collaboration models for improving adolescent SRH: horizontal integration between healthcare and education, integration among governmental and non-governmental organizations, and diagonal integration between community stakeholders and service providers. Findings showed that successful collaborations relied on three processes – trust-building, shared goals, and clear communication. The availability of champions and intermediaries facilitated the collaborations, supported by strong leadership, dedicated funding, and favourable policies. However, resource constraints, conflicting interests, and organizational silos were key constraints.

The collaboration between the health sector and the education sector in establishing school health clubs for ASRH reflects a recognition of the interconnectedness of health and education in influencing adolescent well-being and holistic development [8]. Health and education are interlinked determinants of well-being, and joint efforts contribute to a more comprehensive understanding of adolescents’ needs and challenges. Existing literature emphasizes the significance of school-based interventions, particularly those involving health and education sectors, in delivering comprehensive sexuality education [12, 13].

School health clubs provide a platform to integrate CSE into the educational curriculum, fostering a supportive environment for adolescents to access accurate information about sexual and reproductive health [36]. Establishing health clubs in schools aligns with the literature emphasizing the importance of engaging adolescents as active participants in their health and well-being [37, 38]. Such initiatives not only deliver information but also empower adolescents to take ownership of their sexual and reproductive health. Moreover, integrating health promotion activities within the school setting bridges the gap between formal education and community-based health initiatives, creating a space where both sectors can contribute to positive health outcomes [39]. Finally, the intersectoral collaboration in school health clubs aligns with the understanding that education is a social determinant of health [40].

Finding of collaboration between formal providers and informal providers in delivering SRH services to adolescents aligns with existing literature that recognizes the diverse healthcare landscape and emphasizes the importance of leveraging all available resources to meet the unique needs of this population [41, 42]. Collaboration between formal and informal providers contributes to delivering more comprehensive and accessible SRH services [43, 44]. In many resource-constrained settings, informal providers play a vital role in filling gaps in healthcare delivery. Collaborating with them helps to extend the reach of SRH services, especially in areas where formal healthcare infrastructure may be limited or inaccessible. Informal providers may have established trust within the community and possess cultural competence, making them well-suited to reach and engage adolescents who might face barriers in accessing formal healthcare services [42, 45, 46]. Engaging informal providers in SRH services promotes community engagement because these providers are often local actors who understand the sociocultural context [47].

Collaboration between community leaders and healthcare workers reflects a community-based participatory approach to adolescent SRH. Existing literature emphasizes the importance of involving local leaders and stakeholders in the planning, implementing, and evaluating health interventions, ensuring interventions are culturally competent and community-tailored [48]. Engaging community leaders in SRH activities promotes a sense of ownership and sustainability within the community and helps to reinforce the notion that health is a shared responsibility [49]. Community leaders, acting as intermediaries, can help overcome barriers to healthcare access and facilitate adolescents' engagement in health-promoting activities [50]. Furthermore, collaboration between community leaders and healthcare workers builds trust and credibility within the community. This is crucial for the success of adolescent SRH programs, as trust facilitates open communication and acceptance of healthcare services.

Processes that were used to achieve successful collaborations among stakeholders in the research were trust-building, shared goals, and clear communication. They are consistently identified as key drivers that enhance the effectiveness and sustainability of stakeholder partnerships. Trust among stakeholders fosters open communication, reduces conflicts, and enhances cooperation. When stakeholders have a common purpose (shared goals), it creates a sense of unity and direction. Effective communication helps in setting expectations, resolving misunderstandings, and coordinating activities among stakeholders. It ensures transparency and accountability, essential for building trust and maintaining a productive partnership.

Findings showed that collaborations among stakeholders are influenced by various factors, including the availability of champions, strong leadership, dedicated funding, and policies favouring cross-organizational interaction. Literature emphasizes the critical role of champions in initiating and sustaining collaborative efforts. They often possess the influence, commitment, and skills to mobilize resources, motivate stakeholders, and navigate organizational barriers. Strong leaders provide vision, direction, and support, enabling stakeholders to work together more effectively. Dedicated funding ensures that collaborative initiatives have the necessary financial support to undertake joint activities, implement programs, and sustain operations. Without adequate funding, even well-planned collaborations can falter due to resource constraints, making financial support a critical enabling factor.

A major strength of this qualitative research is the holistic exploration of the perspectives of a diverse range of stakeholders, including policymakers, program managers, formal and informal health providers, and community leaders. Thus, it provides a comprehensive overview of the collaborative landscape and insights into the multi-dimensional nature of intersectoral collaboration. However, the study has limited external validity or generalizability to other regions or settings beyond Ebonyi State. The local context and specific characteristics of Ebonyi State may not be entirely representative of other geographical areas. Moreover, collaboration dynamics are often dynamic and can often change over time. The research captures a snapshot of these dynamics, but ongoing changes or shifts in collaboration structures may not be fully captured.

## Conclusions

Findings from this study highlight the potential of community-embedded interventions to strengthen intersectoral collaboration for the acceptability and adoption of ASRH strategies. The implementation research study sheds light on the various types of collaborations formed, the intricate processes involved in creating and revitalizing these collaborations, and the influential factors that either enabled or constrained these partnerships.

This paper proposes a framework for stakeholder collaboration in adolescent SRH that integrates four themes: (i) Processes that underpin effective stakeholder collaboration—trust-building, shared goals, and clear communication; (ii) Approaches for reviving dormant collaborations—stakeholder engagement, capacity-building, and policy alignment; (iii) Factors that facilitate collaborations—champions and intermediaries, a supportive institutional framework, robust leadership commitment, dedicated funding mechanisms, and a favourable policy environment; (iv) Factors that hinder collaborations—resource constraints, conflicting interests, and organizational silos**.**

## Data Availability

The dataset generated and/or analyzed during this study is contained in the manuscript.

## Abbreviations

ASRH: Adolescent sexual and reproductive health
CHW: Community health worker
FGD: Focus group discussion
IDI: In-depth interview
LGA: Local government area
NDHS: National demographic and health survey
OIC: Officer-in-charge
PPMV: Patent and proprietary medicine vendor
REC: Research and Ethics committee
SRH: Sexual and reproductive health
